# Perinatal Exposure to Glufosinate Ammonium Herbicide Impairs Neurogenesis and Neuroblast Migration through Cytoskeleton Destabilization

**DOI:** 10.3389/fncel.2016.00191

**Published:** 2016-08-09

**Authors:** Ameziane Herzine, Anthony Laugeray, Justyne Feat, Arnaud Menuet, Valérie Quesniaux, Olivier Richard, Jacques Pichon, Céline Montécot-Dubourg, Olivier Perche, Stéphane Mortaud

**Affiliations:** ^1^UMR7355, Centre National de la Recherche ScientifiqueOrleans, France; ^2^Immunologie et Neurogénétique Expérimentales et Moléculaires, Experimental and Molecular Immunology and Neurogenetics, University of OrleansOrleans, France; ^3^Genetics Department, Regional HospitalOrleans, France

**Keywords:** glufosinate ammonium, pesticide, neuroblasts migration, SVZ, neuro-development, cytoskeleton, Autism Spectrum Disorder

## Abstract

Neurogenesis, a process of generating functional neurons from neural precursors, occurs throughout life in restricted brain regions such as the subventricular zone (SVZ). During this process, newly generated neurons migrate along the rostral migratory stream to the olfactory bulb to replace granule cells and periglomerular neurons. This neuronal migration is pivotal not only for neuronal plasticity but also for adapted olfactory based behaviors. Perturbation of this highly controlled system by exogenous chemicals has been associated with neurodevelopmental disorders. We reported recently that perinatal exposure to low dose herbicide glufosinate ammonium (GLA), leads to long lasting behavioral defects reminiscent of Autism Spectrum Disorder-like phenotype in the offspring (Laugeray et al., 2014). Herein, we demonstrate that perinatal exposure to low dose GLA induces alterations in neuroblast proliferation within the SVZ and abnormal migration from the SVZ to the olfactory bulbs. These disturbances are not only concomitant to changes in cell morphology, proliferation and apoptosis, but are also associated with transcriptomic changes. Therefore, we demonstrate for the first time that perinatal exposure to low dose GLA alters SVZ neurogenesis. Jointly with our previous work, the present results provide new evidence on the link between molecular and cellular consequences of early life exposure to the herbicide GLA and the onset of ASD-like phenotype later in life.

## Introduction

Production of neurons is an active ongoing process with 10,000 to 30,000 neurons being produced daily in rodents (Lledo et al., 2006). While the molecular mechanisms involved are not fully understood, the persistence of neurogenesis in adult subventricular zone (SVZ) is currently well-established (Ghashghaei et al., 2007). The SVZ newly generated neurons migrate along the rostral migratory stream (RMS) to the olfactory bulb (OB) where they will be used to replace granule cells and periglomerular neurons (Lois and Alvarez-Buylla, 1994). RMS is characterized by neuroblasts organized into chains continually migrating through an astrocytic tube-like structure called the glial tube (Sun et al., 2010). This ongoing process is tightly regulated through multiple secretory signals including mitogenic factors (i.e., EGF, Notch) within the SVZ, repulsive factors along the RMS (i.e., Slit, Ephrin, Netrin), and chemoattractive factors (i.e., Reelin, Neuregulin) within the OB (Coskun and Luskin, 2002; Whitman and Greer, 2009). In terms of overall brain function, neuroblast migration is of particular relevance to olfactory-based neuronal plasticity and for consequent adapted behavior such as fine-odor discrimination (Gheusi, 2000).

As for all tightly controlled system, any disturbances could dramatically change the outcome of neuroblast generation or migration (Khodosevich et al., 2009; Sun et al., 2010; Young et al., 2011), and so, would likely lead to neurodevelopmental disorders. In line with this, Autism Spectrum Disorders (ASD) are thought to be associated with alterations in neonatal neurogenesis in the SVZ (Kotagiri et al., 2014). Indeed, it was shown that neurobehavioral defects concomitant to cellular SVZ abnormalities were induced by perinatal exposure to methotrexate (Seigers et al., 2015; Hirako et al., 2016).

Many mediators are involved in the homeostasis of SVZ neurogenesis and neuroblast migration but the neurotransmitter glutamate has been shown to be of particular importance in controlling these processes (Di Giorgi-Gerevini et al., 2005; Platel et al., 2007, 2010). The herbicide Glufosinate ammonium (GLA), is the ammonium salt of phosphinothricin (D,L-homoalanin-4-[methyl] phosphinate), an aminoacid structurally related to glutamate and as such is likely to interfere with glutamate signaling. Acute exposure to GLA causes disturbances of the glutamate homeostasis, memory impairments, brain structural modifications, and astrogliosis (Nakaki et al., 2000; Calas et al., 2008, 2016; Meme et al., 2009). Furthermore, we recently showed that GLA has pervasive and harmful effects when administered during the highly sensitive pre- and post-natal periods of brain development (Laugeray et al., 2014). Neurobehavioral tests revealed significant effects of maternal exposure to GLA on offspring's early reflex development, mother-pup communication and affiliative behaviors later in life. We could also show that perinatal exposure to GLA strongly affected offspring's ability to prefer social olfactory cues over non-social ones. This latter alteration led us to assume that the birth/renewal of SVZ—olfactory bulbs neurons (the main system underlying olfactory-based behaviors in rodents) may be compromised by the herbicide. Interestingly, the expression of two genes involved in the regulation of neuroblast proliferation, migration and apoptosis during brain development (Li et al., 2002; Wang et al., 2004), *Pten* and *Peg3*, were dysregulated in GLA-exposed offspring's brain (Laugeray et al., 2014).

Based on these behavioral and gene expression disturbances, we were interested in the present report in investigating whether GLA-induced-neuropathological conditions may be due to neurogenesis defects and alterations in neuroblast homeostasis by using complementary *in vivo* and *ex-vivo* approaches.

## Materials and methods

### Animals and treatments

**Figure 1:**
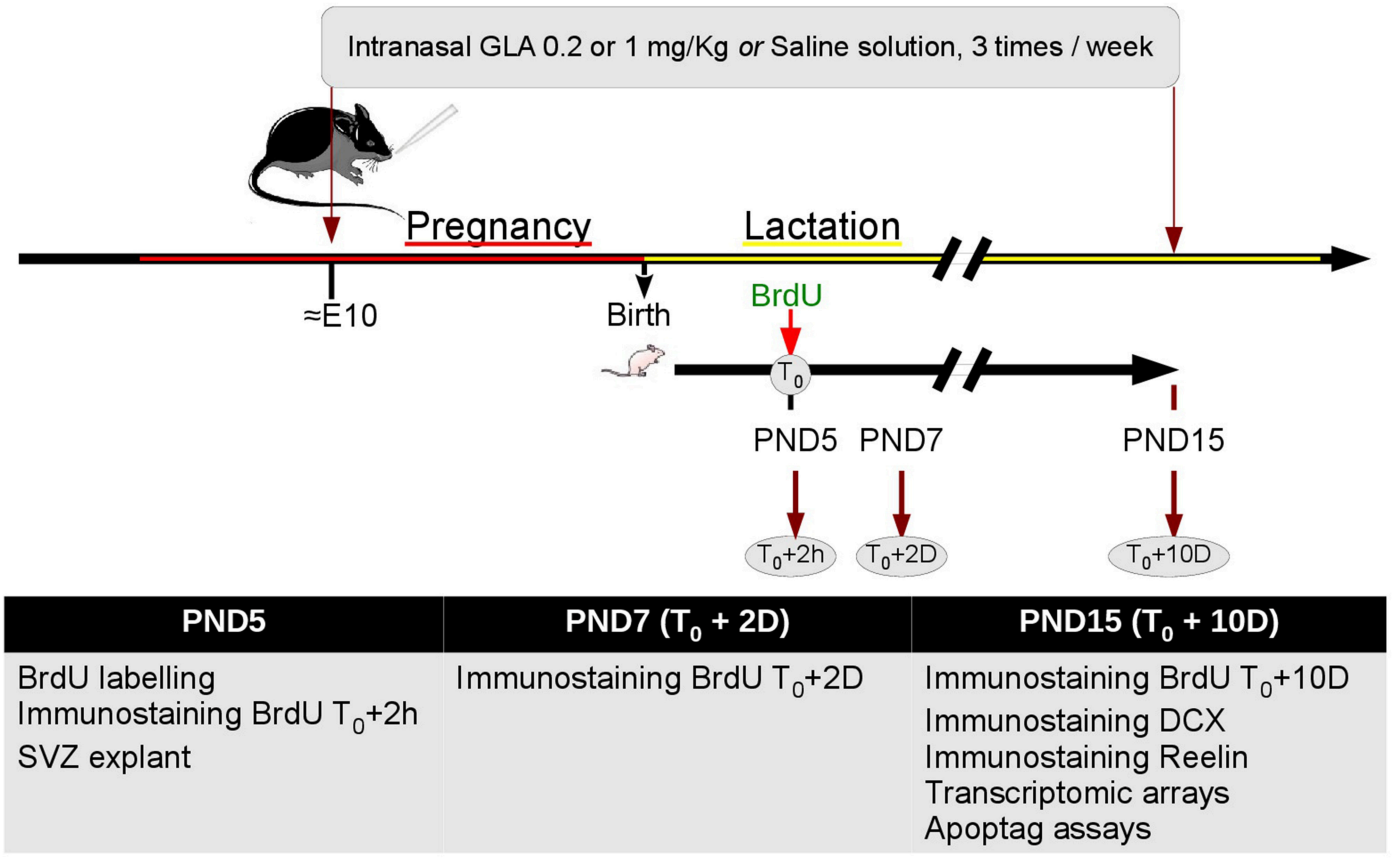
**Study experimental design**. After 2 weeks acclimation female mice were paired with male mice for 5–6 days to mate. Pregnant mice were treated intranasally with either GLA (0.2 or 1 mg/kg) or saline solution. Dams were treated three times a week from embryonic day 10 (E10) to postnatal day 15 (PND15). At PND5, a group of pups were injected with 100 mg/kg BrdU; 2 h (T0+2 h), 48 h (T0+2 D) or 10 days later (T0+10 D), they were euthanized, and the brains processed for immunohistochemistry. Other pups were euthanized at PND5 and brains processed for culture of SVZ explant, or at PND15 and brains processed for transcriptomic arrays.

Seven-week-old female C57Bl/6 mice were purchased from Janvier (Le Genest St Isle, France). All mice were bred and maintained on a 12-h light/dark cycle (lights on from 7:00 a.m. to 7:00 p.m.) with food and water *ad libitum* in a temperature controlled (21 ± 1°C) room in the animal resource facility. After an acclimatization period of 2 weeks, female mice were mated with male C57Bl6 mice also obtained from Janvier (Le Genest St Isle, France) during 5–6 days. Pregnant mice were then isolated and divided in three experimental groups treated intranasally with either GLA (1 or 0.2 mg/kg; PESTANAL®, analytical standard from Sigma–Aldrich) or saline solution (NaCl 0.9%; 10 μl/30 g mouse) as control. These two doses were not only chosen as they were shown to induce bio-behavioral abnormalities in our previous study (Laugeray et al., 2014) but also because they are ~5 to 25 times lower than the EPA approved dose (EPA, 2012). Intranasal exposure was not only chosen as a realistic model of human exposure to volatile toxicants through inhalation but also as it is known that such substances may reach the body via the systemic route without liver interaction (Benson et al., 1999; Amuzie et al., 2008). Therefore, many volatile substances may have deleterious consequences, especially in situation of long-lasting or recurrent exposure to low dose pesticides. Dams were treated during pregnancy and lactation periods three times a week from embryonic day 7-10 (E7-10) to postnatal day 15 (PND15) (Figure 1). Control animals received a comparable dose of 0.9% saline vehicle. Offspring were maintained in same-sex, litter-mate housed cages with *ad libitum* access to food and water. All aspects of animal care and experimentation were in accordance with the European Communities Council directive (2010/63/EU). The Ethics committee approved all animal care and use for this study (Approval C45-234-6).

### General procedure

General procedure is shown in Figure 1. From E10 to PND15, we performed preweaning tests to check developmental consequences of perinatal exposure to GLA. With this aim, we investigated neuroblast migration and proliferation by Bromodeoxyuridine (BrdU) labeling *in vivo* from PND5 to PND15 and *ex vivo* on Matrigel at PND5. Western Blot, Apoptag®, immunochemistry and transcriptomic arrays were performed on PND15 mice brain.

### Cultures of SVZ explants

Brains from 5 day-old C57bl/6 perinatal treated mice (GLA0.2 or GLA1) or control (CTL) were positioned in ice-cold Leibovitz's L-15 medium (GIBCO). The SVZ from the lateral wall was dissected and sectioned using a vibratome (Leica VT1200S) into pieces 50–300 μm in diameter. The explants were mixed with BD Matrigel^™^ Matrix (BD Biosciences) and allowed to solidify in a culture dish (GIBCO). The gel containing the explants was overlaid with 500 μl of Neurobasal medium (GIBCO) containing 10% SVF (Hyclon), B-27 supplement (GIBCO), 0.5 mM L-glutamine (GIBCO), and penicillin–streptomycin antibiotics (GIBCO). Cultures were maintained 3 days *in vitro* (3DIV) in a humidified, 5% CO2, 37°C incubator (RS Biotech).

### BrdU labeling

Bromodeoxyuridine (BrdU, Sigma), commonly used as a mitotic marker, can inform on neuroblast migration. It is well-described that within the RMS, migrating neuroblasts divide only in the initial portion of the RMS, so BrdU+ cells detected in rostral regions must have proliferated in caudal regions or SVZ, making BrdU labeling a strategy to trace cell migration from the SVZ to the OB. This approach allowed us to evaluate the proliferation and the migration of neuroblasts in the RMS. This kind of analysis had the advantage of looking at the entire population of cells being generated. Thus, cells labeled, although not directly identified, are likely to be primarily precursors of olfactory bulb neurons (Ono et al., 1994). Migration of cells from the SVZ to the OB involves several processes: the initial decision to exit the SVZ, the migration of cell along the RMS, and the radial migration of individual cells from the end of the RMS into the glomerular layer of the OB (Peretto et al., 1999).

To evaluate the proliferative and migrating abilities of neuroblasts between the SVZ and the OB, BrdU (100 mg/kg; Figure 1) was administered subcutaneous to PND5 GLA-exposed, as well as controls pups. After 2 h, 2 days or 10 days, brains were harvested, fixed, cryoprotected (see below for details) and 14 μm sections were processed (obtained from a cryostat; Leica) using standard immunofluorescence techniques with BrdU monoclonal antibody Alexa Fluor 488 at 1:1000 dilution, counterstained with DAPI (10 μg/ml, Sigma) and coverslipped with fluoromount. Because of the impossibility to determine the number of nuclei within the RMS and the neuroepithelium (NE), we measured integrated intensity (RawIntDen) of BrdU-labeled cells located in the rostral SVZ, the NE and glomerular layer (Glo) with ImageJ® software (NIH) on raw 32 bits imagery according to ImageJ recommendation (Burgess et al., 2010; McCloy et al., 2014) (Supplementary Figure 2). At least 10 sections per animal and per structure were examined.

### Immunohistochemistry (IHC)

Brains from PND5, 7, and 15 were fixed by immersion in 4% paraformaldehyde (PFA) in 100 mM phosphate buffer, pH 7.4, for 72 h at 4°C and cryoprotected in Tris-buffered saline (TBS) (50 mM Tris-Hcl, pH7.5, 150 mM NaCl) containing 30% sucrose before embedding in Optimum Cutting Temperature (OCT) compound (Sakura Finetek) and frozen in isopentane cooled up to −50°C. Histological coronal sections were mounted onto superfrost+ slides (VWR). Antigen retrieval was performed by incubating the sections in 10 mM sodium citrate solution (pH 9.0) for 30 min in an oven (80°C) (Jiao et al., 1999) and pre-blocked for IHC for 30 min to 1 h in TBS with 0.3% Triton X-100, 10% normal goat serum and 1% bovine serum albumine (BSA). Sections were incubated overnight at 4°C with the primary antibodies. The following antibodies were used: rabbit polyclonal (pAb) anti-DCX (1:500; Abcam, ab77450), rat monoclonal pAb anti-BrdU (1:500; Abcam, ab6326), mouse monoclonal (mAb) anti-Reelin (1:500; Abcam, ab18570) and species-specific secondary antibodies (Abcam). Sections were counterstained with DAPI (10 μg/ml, Sigma), mounted in Fluoromount-G (SouthernBiotech) and images were captured using a fluorescence microscope (DM6000B; Leica Microsystems) powered by Metamorph software. Measurements were performed with Image J.

In the case of concomitant labeling of BrdU, selected sections from BrdU labeled brains was initiated by the pre-treatment of sections with 10 mM sodium citrate solution (pH 9.0) for 30 min in an oven (80°C). Next, 1 N HCl for 10 min at 4°C, then 2N HCL for 30 min at 37°C.

### Immunocytochemistry

Immunological characterization of cells in chains was performed directly in culture dishes. Samples were fixed with 4% PFA solution in TBS (pH 7.4) for 10 min at room temperature and blocked for 20 min in TBS containing 10% normal goat serum, 1% BSA, and 0.2% Triton X-100. Incubation was with primary monoclonal antibodies (rabbit IgG anti-DCX 1:500 dilution; Abcam), carried out 1 h in humidified box at room temperature (RT). The samples were washed with TBS and incubated with species-specific secondary antibodies (anti-IgG Alexa488 Abcam) for 30 min at RT. Samples washed in TBS and counterstained with DAPI (10 μg/ml, Sigma), mounted in Fluoromount-G (SouthernBiotech) and examined with a fluorescence microscope. Images were captured using a Leica DM6000B microscope powered by Metamorph software. Measurements were performed with Image J.

### TUNEL assay

TUNEL assays were conducted with an ApopTag® Red *In Situ* Apoptosis Detection Kit (S7165, EMD Millipore) following the indicated protocol. Briefly, sections were treated as indicated above, fixed in 1% PFA, washed in TBS three times, incubated in equilibration buffer (potassium cacodylate; provided in the kit) for 10 min and incubated with terminal deoxynucleotidyl transferase for 60 min at 37°C. After 10 min in stop buffer (provided in the kit), sections were incubated with anti-digoxigenin conjugate overnight at 4°C. After washing in TBS, sections counterstained with DAPI (10 μg/ml, Sigma), mounted in Fluoromount-G (SouthernBiotech) and examined with a fluorescence microscope (Leica).

### Microscopy and imaging

A conventional fluorescence microscope (Leica DM6000B) was used for the rough inspection of stained sections. Images from stained sections were captured with a digital microscope camera (Leica DFC310 FX) and Metamorph software. Selected fluorescently labeled tissues were analyzed with ImageJ software.

### RNA extraction

RNA extraction from PND15 brain murine tissues was carried out using Trizol reagent (Invitrogen, Carlsbad, CA) following the manufacturer's instructions. Quantity and quality of the total RNA were controlled by Nanodrop spectrophotometer (Nanodrop, Wilmington, DE) and Agilent Bioanalyser (Agilent technologies, Palo Alto, CA) in accordance with manufacturer's instructions. Samples A260/A280 absorbance ratio was greater than 1.8 and 28S/18S rRNA ratio greater than 1.5.

### Affymetrix mice exon 1.0 ST array 1.0 and micro-arrays analyses

Gene expression was tested by the Affymetrix Mice Exon 1.0 ST Array (Affymetrix, Santa Clara, CA). A total of 2 μg of RNA from each brain samples was labeled with reagents from Affymetrix according to manufacturers' instructions. Hybridization cocktails containing 5–5.5 μg of fragmented, end-labeled single-stranded cDNA were prepared and hybridized to GeneChip Mouse Exon 1.0 ST arrays. Arrays were washed, stained and scanned on the Affymetrix Fluidics Station and G7 Affymetrix high-resolution scanner (GCOS 1.3). Affymetrix Expression Console Software^™^ (version 1.0) was used to perform quality assessment. Raw signals were then transformed into “.CEL” files in GCOS software (Affymetrix, Santa Clara, CA). Probe data were generated using the Robust Multi-chip Average with GC-content Background Correction (GCRMA, http://www.bioconductor.org) in Genespring 7 software (Silicon Genetics, Redwood City, CA). This involves background correction, quantile normalization, and summarization of the probe-set values into gene-level expression measurements.

In this study we focus our interest on genes involved in the overall cytoskeleton structure, biogenesis, organization or regulation. Cytoskeleton gene list was obtained from the database GSEA (http://software.broadinstitute.org/gsea/index.jsp), and injected in Genespring 7 to investigate gene deregulation in our experimental groups (GLA0.2 and GLA1). Thus, differentially regulated cytoskeleton genes were determined using a one-way ANOVA analysis and a Benjamini Hochberg False Discovery Rate (FDR) (< 0.05) method for multiple comparison corrections. This statistic protocol was classically used in the literature for analyzing microarrays expression data (Bittel et al., 2007; Perche et al., 2013). Only those genes whose expression changed at least 1.2-fold from the baseline value were selected for downstream analysis. This filter helped maximing the number of genes. PCR quantitative was used to validate the expression arrays, through validation of 45 genes.

Genes that met statistical criteria were analyzed using the DAVID (Database for Annotation, Visualization and Integrated Discovery, Bioinformatics Resources 6.7) for cytoskeleton KEGG pathway visualization.

### Statistical analysis

All data, other than micro array analyses, were analyzed by using parametric procedures: when more than two groups were involved, one-way ANOVA was applied. When appropriate, Dunn's multiple comparison test was performed as *post-hoc* in order to control the false directory rate. Significance was set at *P* < 0.05.

Concerning microarrays, differentially regulated cytoskeleton genes were determined using a one-way ANOVA analysis and a Benjamini Hochberg False Discovery Rate (FDR) (< 0.05) method for multiple comparison corrections.

## Results

### Effect of GLA exposure on subventricular zone structure and neuroblast chain migration

**Figure 2:**
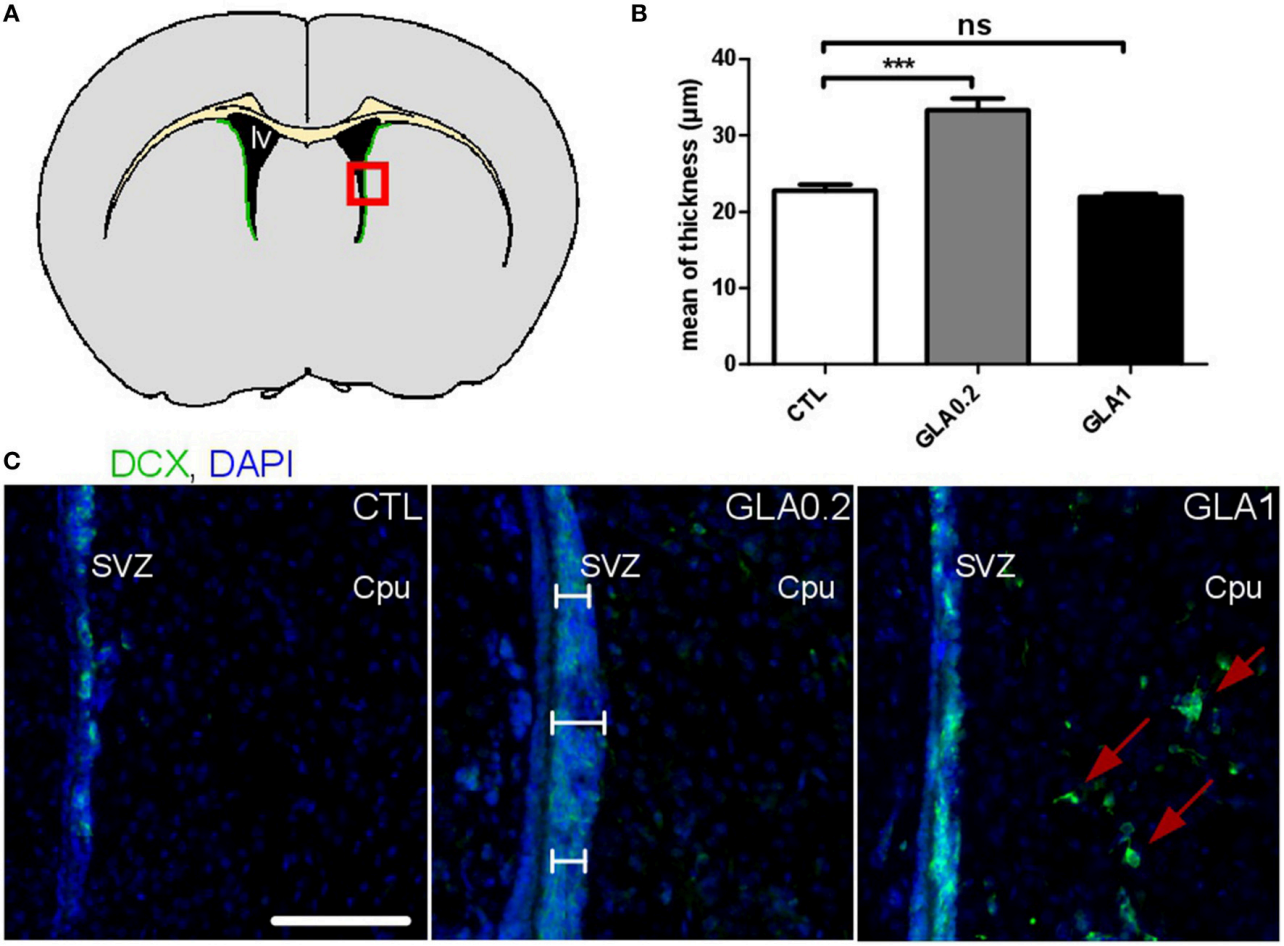
**Effect of perinatal glufosinate ammonium exposure on neuroblast migration along the SVZ**. **(A)** Diagram of a coronal section at SVZ level (green area). Coronal sections (red square) were stained with DCX (doublecortin; nearly exclusive expression in neuroblasts; green staining) and counterstained with DAPI (nuclear blue staining). Three measurements of thickness were carried for each SVZ (2 SVZ for each coronal section). Three coronal sections were analyzed per animal (Bregma 1.145; 0.745; 0.245); the mean of all values represents one mice. **(B)** The SVZ thickness of GLA0.2 exposed mice (*n* = 5) was significantly increased compared to CTL mice (*n* = 5). No difference was found in GLA1 exposed mice (*n* = 6). **(C)** Sections from GLA0.2 mice at PND15 display a more extensive SVZ thickness than CTL. SVZ thickness from GLA1 mice was similar to CTL but with ectopic migration of neuroblasts outside the SVZ (red arrows). Scale bar 100 μm. Each value represents the mean ± SEM (^***^p < 0.001). lv, lateral ventricle; Cpu, Caudate putamen.

**Figure 3:**
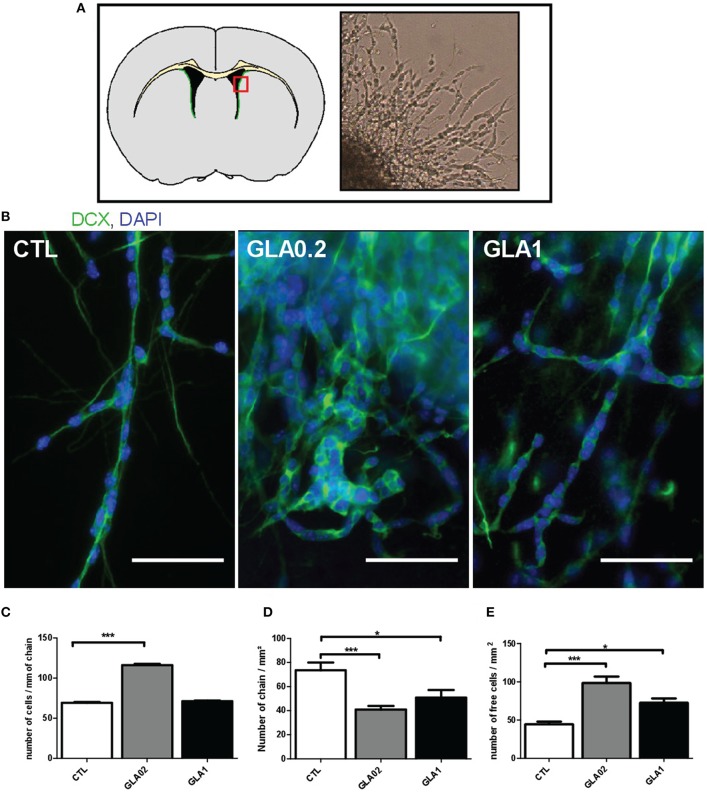
***Ex vivo* culture of SVZ explant from perinatal GLA exposed mice**. Culture on Matrigel of explants from the SVZ of brains from 5 day-old pups treated perinatal with GLA0.2 (*n* = 11), GLA1 (*n* = 7) or control (CTL, *n* = 10) was performed for 3 days. We analyzed 6 to 10 explants per animal; the mean of all values represent one mice. **(A)** The top panel shows a diagram of a coronal section at the SVZ (green area) and the explants were micro-dissected in the party designated by the red square. The photograph on the right of this panel was obtained with an inverted microscope of culture at 3 day *in vitro*. The morphological appearance of neuroblasts and formed chains were investigated by DCX immunocytology to mark neuroblasts (green) and nuclei with DAPI (blue) **(B)**. The results show classical bipolar spindle-shaped cells in contact with each other in CTL. Unlike GLA0.2 GLA1 show extensions and ramifications of the growth cone, a loss of bipolarity and abnormal appearance and compact chains. The number of cells per unit distance was measured in formed chains and show a significant increase in this number in GLA0.2 highlighting aggregation **(C)**. Number of chains formed **(D)** or individual cells **(E)** were counted. The number of chains formed in explants exposed mice to GLA0.2 and GLA1 is significantly lower compared to the CTL. Therefore, the number of isolated free cells is significantly higher in exposed mice. These results show the difficulty of neuroblasts to migrate and form chains. Each value is represented by the mean ± SEM (^*^p < 0.05; ^***^p < 0.001). Scale bar 100 μm.

With the aim to investigate the effect of GLA exposure on the SVZ, we first carried out morphological measurements of SVZ thickness in PND15 CTL and GLA-exposed pups (Figure 2A). GLA0.2-exposed offspring displayed a 46.9% increased SVZ thickness compared to control mice (Figure 2B). This increase was associated with an increased number of DCX-positive neuroblasts in the SVZ (Figure 2C). Interestingly no difference in SVZ thickness was observed in GLA1-exposed offspring (Figures 2B,C). However, we observed that GLA1 treatment promoted ectopic migration of neuroblasts to the surrounding brain regions as, DCX^+^ cells were observed in the caudate putamen (Cpu). Then we characterized the effect of GLA *ex vivo* by using SVZ explants and verified whether neuroblast morphology and/or migration were altered. CTL explants were made of individual long chains of DCX+ cells, extending from the SVZ while explants collected from GLA-treated pups were quite different as chain morphology was completely altered (Figure 3). Indeed, SVZ explants coming from GLA0.2 pups displayed chains with a compacted morphology compared to CTL. In explants from GLA1-exposed pups, chains of neuroblast were similar to those of CTL explants in terms of thickness but seemed to be cell-enriched. We quantified this phenomenon by measuring the number of nucleus per chain length. We clearly observed an increase in cell number in explants coming from GLA-exposed pups (Figure 3C). Moreover, in explants from GLA1 pups, migrating cells displayed abnormal morphology with (1) extension and branching of the growth cone, (2) abnormally long dendrites, and (3) overall loosing of their bipolarity (Supplementary Figure 3). These data were corroborated by the measurement of the number of chains and the number of individual cells per surface unit. Indeed, there was a significant decrease of the number of chains/mm^2^ for GLA0.2 and GLA1 explants compared to CTL. Further, there was a significant increase in cells migrating individually in the GLA0.2 and GLA1 explants compared to CTL. The effect seemed to be more pronounced at the lowest dose of GLA (Figures 3D,E).

### Perinatal GLA exposure impairs cell proliferation in the sub-ventricular zone and migration toward the olfactory bulb

To verify whether GLA exposure was responsible for changes in the temporal dynamics of neuroblast migration from the SVZ to the OB, BrdU+ cells were determined in the rostral region of the SVZ, the NE and the Glo of the olfactory bulbs at several timepoints post-BrdU injection: 2 h (T0+2 h), 2 days (T0+2 D) or 10 days (T0+10 D) after BrdU injection. Actually, each point gave us information on the location of BrdU-incorporated migrating cells at 5, 7, and 15 days post-natal.

**Figure 4:**
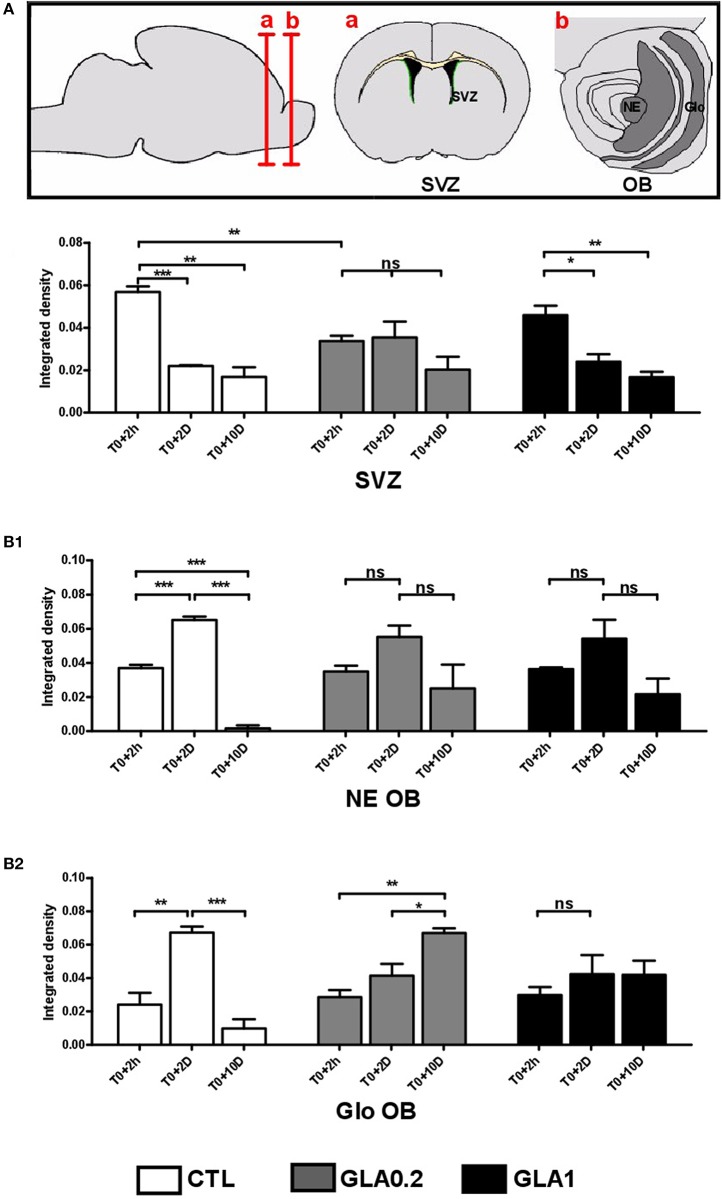
**BrdU labeling reveals GLA-induced alterations of neuroblast migration *in vivo.*** The top panel shows a diagram of coronal sections at the SVZ and OB, the ventricular walls are colored in green. The BrdU labeling intensity was measured in mouse brain coronal sections of CTL (*n* = 3), GLA0.2 (*n* = 3) and GLA1 (*n* = 3) previously injected with BrdU at PND5 and sacrificed after 2 h (T0+2 h) or 2 days (T0+2 D) or 10 days (T0+10 D). The density of BrdU is measured around the SVZ **(A)** or around the OB **(B)** in the neuro-epithelium (NE) **(B1)** and in the glomerular layer (Glo) **(B2)**. At T0+2 h, the BrdU density represents the rate of cell proliferation. The results show a decrease of proliferation only in GLA0.2 animals at the SVZ. No proliferation differences were detected at the OB. Measurements performed at 48 h after BrdU injection (T0+2 D) and compared to those performed at T0+2 h indicate cell movements between the SVZ to the OB in CTL animals. At the SVZ, a decrease of intensity is noted in CTL and GLA1 but not in GLA0.2 exposed animals. In the same way, there is an increase of the BrdU density only in CTL OB. We note here that neuroblasts had difficulties to reach the OB in exposed animals to GLA. At T0+10 D, the fluorescence density of BrdU is lowest and similar in all groups in the SVZ. However, at the OB, the fluorescence density of BrdU is greater in GLA0.2 and GLA1 exposed animals than in CTL. Control, white bar chart, GLA0.2, gray bar chart; GLA1, black bar chart. Each value is represented by the mean ± SEM (^*^p < 0.05; ^**^p < 0.01; ^***^p < 0.001).

**Figure 5:**
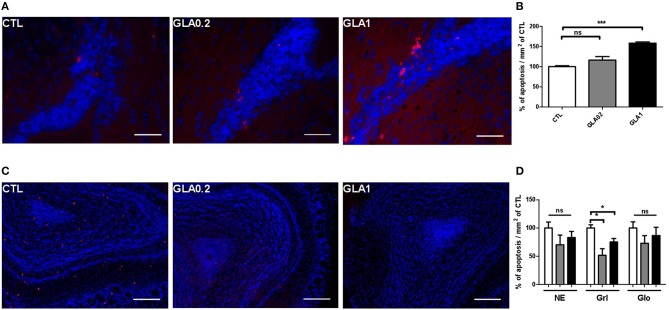
**Apoptotic cells labeling in SVZ and in the olfactory bulb**. Apoptag® immunostaining of coronal sections of CTL (*n* = 3), GLA0.2 (*n* = 3) or GLA1 (*n* = 3) in the SVZ **(A)** and in the OB **(C)** showing a dose effect of GLA in the increase of apoptotic cells in the SVZ of exposed mice **(B)**. Unlike to SVZ, we show a significant decrease of the number of apoptotic cells in the granular layer (Grl) of exposed mice **(D)**. No difference was found in neuro-epithelium (NE) and in glomerular layer (Glo). Each value represents the mean ± SEM (^*^p < 0.05, ^***^p < 0.001). Scale bar 100 μm.

Consistent with previous reports (Luo et al., 2006), BrdU+ nuclei were found throughout the lateral portions of the SVZ (data not shown) at T0+2 h (PND5). Quantitative analysis of BrdU labeling in the SVZ revealed a significant decrease in cell proliferation in GLA0.2-exposed mice compared to CTL while no effect was observed in GLA1-exposed mice (Figure 4A). In the NE and Glo, the intensity of BrdU+ cells was similar in the three groups. At T0+2 D (PND7), in CTL group, about half of the BrdU labeled cells reached the NE while a few remained at the rostral SVZ. Interestingly, a third of the BrdU-labeled SVZ level observed at T0+2 h remained labeled at T0+10 D, suggesting that they incorporated BrdU in or near to their final mitosis. At that time, the SVZ-NE-Glo level of BrdU+ dramatically changed in CTL animals (Figure 4) reflecting the migration of cells toward and into the olfactory bulb. In GLA0.2-exposed mice, this process appeared to be delayed. Indeed, BrdU levels at T0+2 D remained similar to those found in the SVZ at T0+2 h for GLA0.2 exposed mice. This finding suggested that cells could not leave the SVZ with an adapted timing to reach their final location, the OB. The fact that about two-thirds of the BrdU^+^ cells was located in the SVZ at T0+10 D not only strengthened this hypothesis (Figure 4B1) but also indicated that, in GLA0.2-exposed-pups, most of the migrating cells stayed close to their final division location for at least 7–10 days. In GLA1-exposed pups, the temporal dynamics of neuroblast migration was differently altered as, like in CTL pups, BrdU-labeling was significantly decreased in the SVZ at T0+2 D. This result indicated that cells were able to leave the SVZ to reach the OB. However, there was no significant increase of BrdU+ cells in NE or OB at T0+2 D contrary to what we observed in the CTL group (Figures 4B1,B2). To verify whether apoptosis may explain GLA-induced changes in neuroblast migration dynamics, we quantified apoptotic cells within the SVZ at PND15 and we observed a significant increase of apoptotic cells at the highest dose of GLA while no change was noticed at the lowest one (Figures 5A,B). Such a phenomenon suggested that apoptotic processes might be involved in GLA1-induced migration defects, and subsequently explain the decreased arrival of BrdU^+^ cells in NE at T0+2 D (Figures 5A,B), while other mechanisms might be at work in GLA0.2-exposed pups.

At T0+10 D, we observed a drastic decrease of BrdU+ cells in NE and Glo in CTL animals (Figures 4B1,B2). Such a phenomenon is normal as many migrating cells die by apoptosis while only a small percentage of these cells reach the OB and integrates local circuits (Khodosevich et al., 2009). In GLA0.2-exposed mice, BrdU+ cells continued to increase at T0+10 D in the OB, while in GLA1-exposed pups, the number of BrdU+ cells remained unchanged (Figure 4B2). Concomitantly, apoptosis analysis within the OB revealed a clear decrease of apoptotic cell number in the granular layer in both GLA0.2 and GLA1-exposed pups compared to CTL even if the latter group was minor affected. No changes were observed in NE and Glo (Figures 5C,D).

### Anatomical alterations of mitral layer structure after GLA exposure

**Figure 6:**
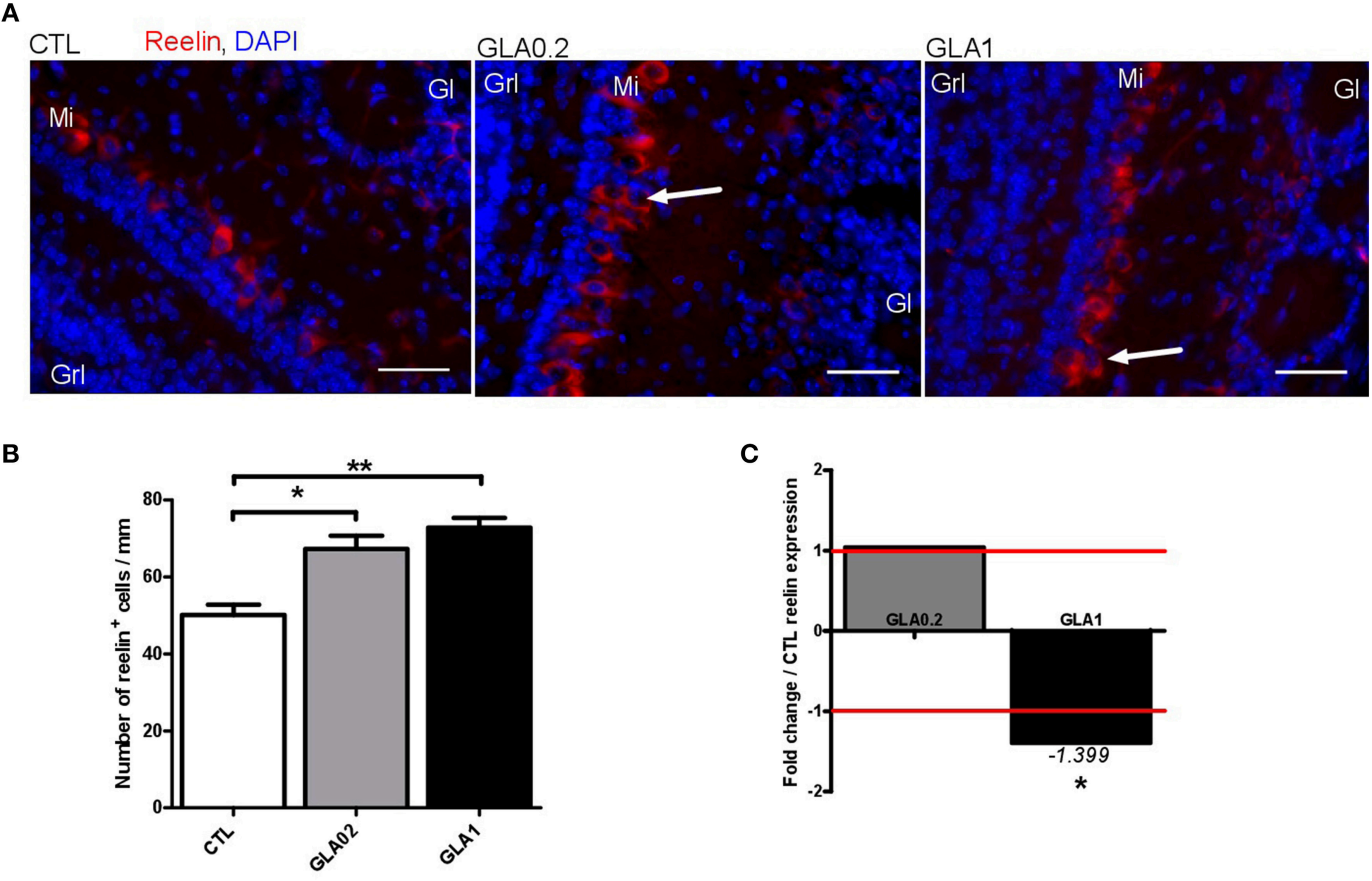
**Effect of perinatal glufosinate ammonium exposure on the mitral cells in the OB**. **(A)** In the OB, four coronal sections per mice were stained with Reelin (nearly exclusive expression in mitral cells; red staining) and counterstained with DAPI (nuclear staining; blue staining) [CTL (*n* = 3), GLA0.2 (*n* = 3), GLA1 (*n* = 3)]. **(B)** The number of mitral cells were determined within the mitral layer. Sections from GLA exposed mice display higher number of mitral cells than CTL. The number of Reelin+ cells/mm of mitral layer is significantly increased in GLA0.2 and GLA1 mice compared to CTL mice. **(C)** mRNA expression levels of reelin on whole brain measured by qPCR. No difference in expression between GLA0.2 (*n* = 8) and CTL (*n* = 8), however Reelin is under expressed in GLA1 (*n* = 8) compared to the CTL. Each value represents the mean ± SEM (^*^p < 0.05; ^**^p < 0.01). Scale bar 50 μm. Mi, mitral layer; Grl, granular layer; Gl, Glomerular layer.

In control mice, reelin immunohistological staining showed two distinct staining regions clearly expressing reelin in the OB—the glomerular (Glo) and mitral cell layers (MCL) (Figure 6). Reelin expression outlined the glomeruli, and was found at high levels in the MCL. In GLA-exposed mice, irrespective of the dose, mitral cells appeared disorganized and the MCL slightly expanded compared to CTL mice (arrows in Figure 6), suggesting defects of neuronal lamination (Supplementary Figure 1). The other layers appeared undisturbed. In normal OB, the mitral cell bodies were located directly above the Granular cell layer (GrL) and were oriented radially with their primary dendrites projected directly toward the Glo. As shown in Figure 6, GLA-exposed mice displayed several differences from this normal organization: mitral cell bodies were not located immediately above the GrL and were orientated radially. Furthermore, immunohistology showed an increase of reelin+ cell number both after GLA0.2 and GLA1 perinatal exposure. In addition, analysis of whole brain transcriptomic data highlighted that GLA1-exposed group showed ~40% lower *reelin* gene expression compared to CTL.

### Alteration of cytoskeleton regulation after GLA exposure

Since based on our histological analysis showing that GLA exposure altered neuroblast migration and proliferation as well as *Pten* expression (Laugeray et al., 2014), we decided to explore cytoskeleton gene expression by transcriptomic.

**Figure 7:**
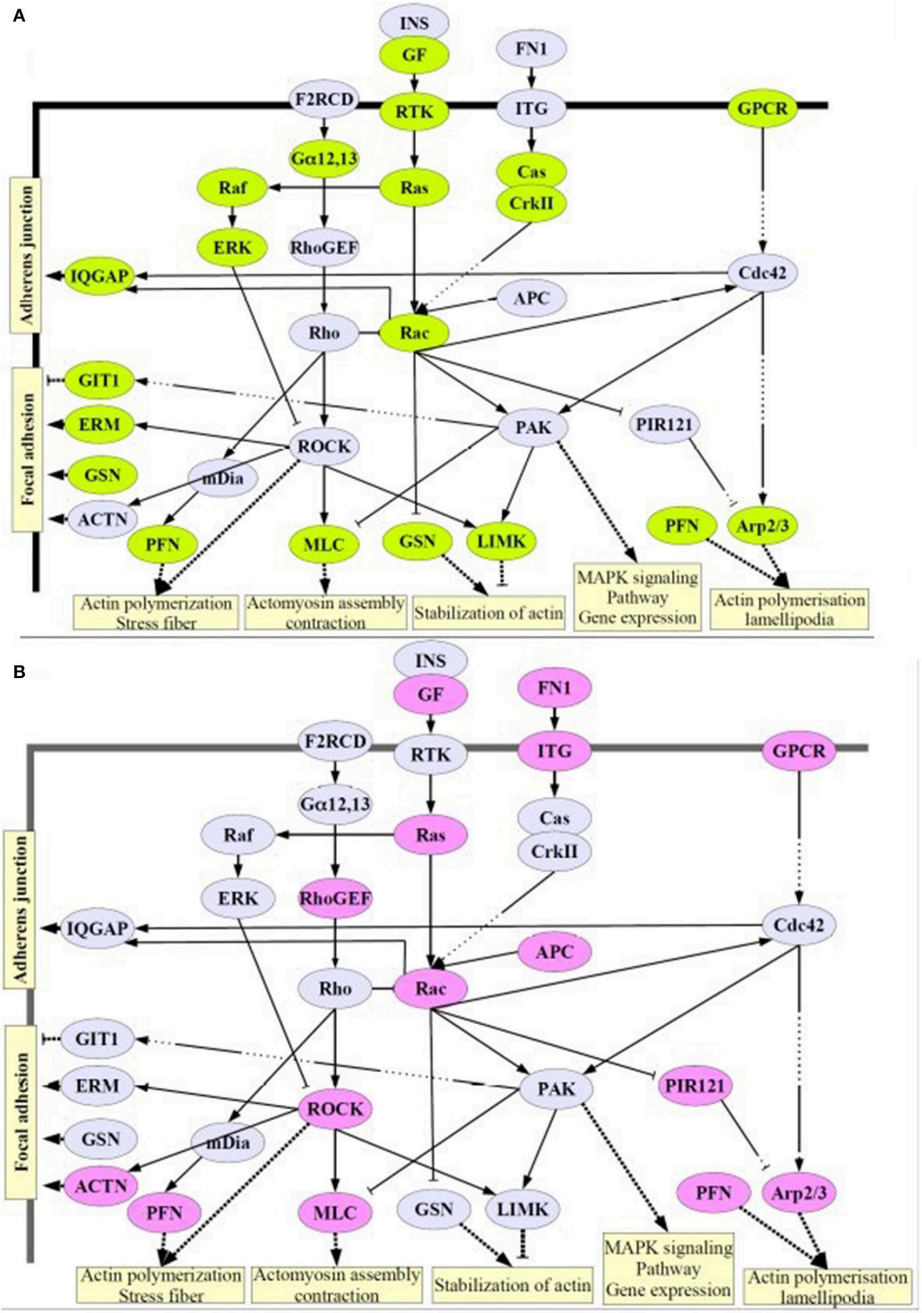
**Signaling pathways involving the cytoskeleton remodeling**. Here we show proteins whose expression is deregulated in GLA0.2- **(A)** and GLA1-exposed pups **(B)**. Significant number of deregulated genes after perinatal exposure to GLA, involved in cell migration and cytoskeleton dynamics can cause problems at cellular levels of migration and adhesion and even vesicular transport in the cell. Figure from David Software database and adapted by S. Mortaud. Differentially regulated cytoskeleton genes were determined using a one-way ANOVA analysis and a Benjamini Hochberg False Discovery Rate (FDR) (< 0.05) method for multiple comparison corrections.

For this purpose, we focused on gene expression levels of cytoskeleton pathway and selected from GSEA database a list of 494 genes involved in cytoskeleton structure, biogenesis, organization, or regulation. Among this list, one-way ANOVA followed by Benjamini Hochberg multiple testing correction demonstrated that 371 genes were significantly deregulated by GLA parental exposure. Among the 371 genes, we showed that 122 genes were deregulated with at least 1.2-fold change (FC1.2) in GLA0.2 and/or GLA1-exposed pups compared to CTL (Table 1). Interestingly, 60 genes (10 Up, 50 Down) were deregulated in GLA0.2-exposed pups whereas 73 genes (37 Up, 36 Down) were affected in GLA1-exposed pups. Only 11 genes, involved in actin cytoskeleton regulation (KEGG pathway, DAVID, Bioinformatics Resources 6.7), were commonly deregulated by both GLA perinatal treatments (Table 1–lines in gray, and Figure 7). However, the deregulation way (up or down-regulated) of these 11 genes were dependent of GLA dose exposure. Indeed, in GLA0.2-exposed pups, 10 out of 11 genes were down-regulated (Table 1, lines in gray) whereas these same genes were up-regulated in GLA1-exposed pups. Only one gene, *Fscn1*, was down-regulated in both conditions.

**Table 1:** **Cytoskeleton-deregulated genes after perinatal GLA exposure**.

**Transcripts Cluster Id**	**FC ([GLA0.2] vs. [CTL])**	**Log FC ([GLA0.2] vs. [CTL])**	**FC ([GLA1] vs. [CTL])**	**Log FC ([GLA1] vs. [CTL])**	**Gene Description**	**Gene Symbol**
6839743	–1.20	–0.27	1.06	0.09	v-crk sarcoma virus CT10 oncogene homolog (avian)-like	*Crkl*
6970635	–1.20	–0.27	–1.01	–0.02	Related RAS viral (r-ras) oncogene homolog 2	*Rras2*
6791641	–1.62	–0.70	1.10	0.14	Glial fibrillary acidic protein	*Gfap*
6905321	–1.59	–0.66	1.10	0.14	Profilin 2	*Pfn2*
6964244	–1.45	–0.54	1.11	0.15	Mitogen-activated protein kinase 3	*Mapk3*
6942751	–1.44	–0.52	–1.18	–0.23	Guanine nucleotide binding protein, alpha 12	*Gna12*
6771558	–1.43	–0.51	1.13	0.17	Dynactin 2	*Dctn2*
6819974	–1.42	–0.51	–1.07	–0.10	Neurofilament, light polypeptide	*Nefl*
6787176	–1.42	–0.50	1.01	0.02	nucleophosmin 1	*Npm1*
6985399	–1.41	–0.50	1.13	0.18	Breast cancer anti-estrogen resistance 1	*Bcar1*
6942654	–1.40	–0.48	–1.13	–0.18	Platelet derived growth factor, alpha	*Pdgfa*
6970139	–1.38	–0.47	1.05	0.07	ADP-ribosylation factor interacting protein 2	*Arfip2*
6871078	–1.35	–0.43	1.13	0.17	Kinesin light chain 2	*Klc2*
6929817	–1.35	–0.43	–1.11	–0.15	Fibroblast growth factor receptor 3	*Fgfr3*
6795784	–1.33	–0.41	–1.04	–0.05	ADP-ribosylation factor 6 | predicted gene 9887	*Arf6|Gm9887*
6941780	–1.33	–0.41	1.04	0.06	Ras homolog gene family, member f | transmembrane protein 120B	*Rhof|Tmem120b*
6789344	–1.33	–0.41	–1.07	–0.10	Fibroblast growth factor 11 | sperm maturation 1	*Fgf11|Spem1*
6804486	–1.32	–0.41	–1.07	–0.10	Guanosine diphosphate (GDP) dissociation inhibitor 2	*Gdi2*
6837144	–1.30	–0.38	–1.06	–0.08	Platelet derived growth factor, B polypeptide	*Pdgfb*
6850749	–1.30	–0.37	1.15	0.20	Tubulin-specific chaperone C	*Tbcc*
6876380	–1.29	–0.37	–1.01	–0.01	Gelsolin	*Gsn*
6870125	–1.29	–0.37	–1.02	–0.03	Internexin neuronal intermediate filament protein, alpha | 5'-nucleotidase, cytosolic II	*Ina|Nt5c2*
6918036	–1.29	–0.37	1.11	0.16	Capping protein (actin filament) muscle Z-line, beta	*Capzb*
6838136	–1.27	–0.34	–1.04	–0.06	Twinfilin, actin-binding protein, homolog 1 (Drosophila)	*Twf1*
6965786	–1.27	–0.34	1.01	0.01	MAP/microtubule affinity-regulating kinase 4	*Mark4*
6848568	–1.27	–0.34	1.09	0.12	Ezrin	*Ezr*
6782572	–1.26	–0.34	–1.04	–0.05	G protein-coupled receptor kinase-interactor 1	*Git1*
6984416	–1.26	–0.33	1.16	0.21	Nudix (nucleoside diphosphate linked moiety X)-type motif 21	*Nudt21*
6838392	–1.25	–0.32	1.13	0.17	Rho family GTPase 1	*Rnd1*
6791204	–1.25	–0.32	–1.05	–0.07	SRC kinase signaling inhibitor 1	*Srcin1*
6784244	–1.25	–0.32	1.15	0.20	Tubulin, gamma 1 | tubulin, gamma 2	*Tubg1|Tubg2*
6790585	–1.24	–0.31	1.04	0.05	Dynein light chain LC8-type 2	*Dynll2*
6942909	–1.24	–0.31	–1.13	–0.17	RAS-related C3 botulinum substrate 1 | diacylglycerol lipase, beta	*Rac1|Daglb*
6959452	–1.23	–0.30	–1.09	–0.12	Sirtuin 2 (silent mating type information regulation 2, homolog) 2 (*S. cerevisiae*)	*Sirt2*
6781456	–1.22	–0.29	–1.07	–0.10	Lethal giant larvae homolog 1 (Drosophila)	*Llgl1*
6843886	–1.22	–0.29	1.04	0.06	Nucleotide binding protein 1 | family with sequence similarity 18, member A	*Nubp1|Fam18a*
6881787	–1.22	–0.29	–1.03	–0.04	Destrin	*Dstn*
6774794	–1.21	–0.28	1.13	0.17	Cyclin-dependent kinase 1	*Cdk1*
6942379	–1.21	–0.27	1.05	0.07	LIM-domain containing, protein kinase	*Limk1*
6933073	1.21	0.27	–1.16	–0.22	Polycystic kidney disease 2	*Pkd2*
6897556	1.21	0.27	–1.08	–0.11	Transient receptor potential cation channel, subfamily C, member 4	*Trpc4*
6899092	1.21	0.28	1.00	0.01	IQ motif containing GTPase activating protein 3	*Iqgap3*
6811117	1.22	0.28	1.03	0.04	Tubulin-specific chaperone E	*Tbce*
7013165	1.24	0.31	1.20	0.26	Fibroblast growth factor 16	*Fgf16*
6877080	1.26	0.33	–1.19	–0.25	Rap1 interacting factor 1 homolog (yeast) | nebulin	*Rif1|Neb*
6919300	1.27	0.34	1.03	0.04	Moloney sarcoma oncogene	*Mos*
7019970	1.28	0.36	–1.19	–0.25	Doublecortin	*Dcx*
6783259	1.29	0.36	1.08	0.11	Tubulin, delta 1	*Tubd1*
7011964	1.35	0.43	1.03	0.04	Arginine vasopressin receptor 2 | Rho GTPase activating protein 4	*Avpr2|Arhgap4*
6778528	–1.20	–0.27	1.28	0.36	YKT6 homolog (*S. cerevisiae*)	*Ykt6*
6949084	–1.21	–0.28	1.44	0.52	Actin related protein 2/3 complex, subunit 4	*Arpc4*
6790294	–1.23	–0.30	1.32	0.40	Chemokine (C-C motif) ligand 3	*Ccl3*
6934130	–1.23	–0.30	1.38	0.46	Actin related protein 2/3 complex, subunit 3	*Arpc3*
6882627	–1.26	–0.34	1.48	0.56	Myosin, light polypeptide 9, regulatory	*Myl9*
6879087	–1.29	–0.36	1.20	0.27	Cholinergic receptor, muscarinic 4	*Chrm4*
6935370	–1.29	–0.37	–1.20	–0.26	Fascin homolog 1, actin bundling protein (Strongylocentrotus purpuratus)	*Fscn1*
6894640	–1.30	–0.38	1.36	0.44	Myosin, light polypeptide 9, regulatory	*Myl9*
6789444	–1.31	–0.39	1.39	0.47	Profilin 1	*Pfn1*
6957763	–1.34	–0.43	1.45	0.53	Rho, GDP dissociation inhibitor (GDI) beta	*Arhgdib*
6864837	–1.53	–0.62	1.25	0.32	fibroblast growth factor 1	*Fgf1*
6969916	–1.00	0.00	–1.20	–0.26	Nuclear mitotic apparatus protein 1	*Numa1*
6884986	1.00	0.00	–1.24	–0.31	Nebulette	*Nebl*
6748525	1.06	0.08	–1.57	–0.65	Dystonin | RIKEN cDNA D630036G22 gene	*Dst|D630036G22Rik*
6928487	1.06	0.09	–1.42	–0.50	A kinase (PRKA) anchor protein (yotiao) 9	*Akap9*
6859935	1.13	0.17	–1.40	–0.49	Adenomatosis polyposis coli	*Apc*
6928889	1.14	0.18	–1.40	–0.48	Piccolo (presynaptic cytomatrix protein)	*Pclo*
6768609	1.02	0.03	–1.39	–0.47	Ankyrin 3, epithelial	*Ank3*
6872616	1.13	0.18	–1.39	–0.47	Protein kinase, cGMP-dependent, type I	*Prkg1*
6886244	1.16	0.21	–1.38	–0.47	Low density lipoprotein-related protein 1B (deleted in tumors) | RAN, member RAS oncogene family	*Lrp1b|Ran*
7012305	1.17	0.23	–1.37	–0.45	Dystrophin, muscular dystrophy	*Dmd*
6981190	–1.02	–0.02	–1.34	–0.43	Hook homolog 3 (Drosophila)	*Hook3*
6759621	–1.11	–0.15	–1.33	–0.41	Fibronectin 1	*Fn1*
6781941	–1.03	–0.05	–1.33	–0.41	Myosin, heavy polypeptide 10, non-muscle	*Myh10*
6804898	1.17	0.23	–1.33	–0.41	Lysosomal trafficking regulator	*Lyst*
6801500	1.16	0.21	–1.32	–0.40	Ninein	*Nin*
6787743	–1.05	–0.08	–1.31	–0.39	Cytoplasmic FMR1 interacting protein 2	*Cyfip2*
6903711	1.04	0.05	–1.31	–0.38	Neuroligin 1	*Nlgn1*
6863301	1.15	0.20	–1.30	–0.38	Rho-associated coiled-coil containing protein kinase 1	*Rock1*
6828492	1.19	0.25	–1.29	–0.37	RPTOR independent companion of MTOR, complex 2	*Rictor*
6947558	1.17	0.22	–1.29	–0.37	Alstrom syndrome 1 homolog (human)	*Alms1*
6941813	–1.09	–0.12	–1.27	–0.35	CAP-GLY domain containing linker protein 1 | hypothetical protein LOC100503214	*Clip1|LOC100503214*
6868728	–1.10	–0.14	–1.27	–0.34	Amyloid beta (A4) precursor protein binding, family A, member 1	*Apba1*
6782776	1,12	0.16	–1.26	–0.33	Neurofibromatosis 1	*Nf1*
6975861	1.12	0.17	–1.26	–0.33	Sorbin and SH3 domain containing 2 | RIKEN cDNA D330022K07 gene | hypothetical LOC100503324	*Sorbs2|D330022K07Rik|LOC100503324*
6994954	–1.07	–0.10	–1.25	–0.32	Rho guanine nucleotide exchange factor (GEF) 12	*Arhgef12*
6836973	–1.08	–0.12	–1.25	–0.32	Myosin, heavy polypeptide 9, non-muscle	*Myh9*
6975701	1.06	0.08	–1.25	–0.32	Pericentriolar material 1	*Pcm1*
6969903	–1.02	–0.03	–1.23	–0.30	Nuclear mitotic apparatus protein 1	*Numa1*
6951276	1.06	0.08	–1.22	–0.29	Bicaudal D homolog 1 (Drosophila)	*Bicd1*
6875722	–1.17	–0.22	–1.22	–0.29	ATP-binding cassette, sub-family A (ABC1), member 2	*Abca2*
6795451	1.09	0.13	–1.22	–0.29	Pinin	*Pnn*
6857810	1.04	0.05	–1.21	–0.27	Leucine-rich PPR-motif containing	*Lrpprc*
6796380	1.20	0.26	–1.20	–0.27	actinin, alpha 1 | striamin	*Actn1|Strm*
6758995	1.07	0.10	–1.20	–0.27	Amyotrophic lateral sclerosis 2 (juvenile) homolog (human) | membrane protein, palmitoylated 4 (MAGUK p55 subfamily member 4)	*Als2|Mpp4*
6876154	–1.05	–0.06	–1.20	–0.26	c-abl oncogene 1, non-receptor tyrosine kinase	*Abl1*
6916557	–1.01	–0.01	1.21	0.27	IAP promoted placental gene | transmembrane protein 69	*Ipp|Tmem69*
6839552	–1.19	–0.25	1.21	0.28	Nuclear distribution gene E homolog 1 (A nidulans)	*Nde1*
6887836	–1.03	–0.04	1.21	0.28	WAS/WASL interacting protein family, member 1	*Wipf1*
6880587	–1.17	–0.23	1.21	0.28	HAUS augmin-like complex, subunit 2	*Haus2*
6843088	1.12	0.16	1.21	0.28	Superoxide dismutase 1, soluble	*Sod1*
6946800	1.11	0.15	1.21	0.28	Superoxide dismutase 1, soluble	*Sod1*
6986677	–1.16	–0.22	1.22	0.28	Platelet-derived growth factor, D polypeptide	*Pdgfd*
6776507	1.09	0.13	1.23	0.30	RIKEN cDNA 4930430F08 gene | centrosomal protein 290	*4930430F08Rik|Cep290*
6858126	–1.03	–0.05	1.23	0.30	Karyopherin (importin) alpha 2	*Kpna2*
6769179	–1.19	–0.25	1.24	0.31	Calponin 2	*Cnn2*
7015993	1.11	0.16	1.24	0.32	ubiquitously expressed transcript	*Uxt*
6899767	–1.05	–0.08	1.25	0.32	Integrin, alpha 10 | peroxisomal biogenesis factor 11 beta	*Itga10|Pex11b*
6835329	1.13	0.18	1.26	0.33	Actin-binding Rho activating protein	*Abra*
6864748	1.06	0.08	1.26	0.33	Ubiquitously expressed transcript	*Uxt*
6988603	–1.14	–0.18	1.26	0.34	Thymus cell antigen 1, theta	*Thy1*
6998165	–1.14	–0.19	1.27	0.35	Mitochondrial ribosomal protein S22 | capping protein (actin filament) muscle Z-line, alpha 1	*Mrps22|Capza1*
6960328	–1.19	–0.25	1.27	0.35	Harvey rat sarcoma oncogene, subgroup R	*Rras*
6839334	–1.13	–0.18	1.28	0.36	Nucleotide binding protein 1	*Nubp1*
6792994	–1.04	–0.06	1.29	0.36	Profilin family, member 4	*Pfn4*
7017603	1.00	0.01	1.29	0.36	N(alpha)-acetyltransferase 10, NatA catalytic subunitNalpha acetyltransferase 10 | Rho GTPase activating protein 4	*Naa10|Arhgap4*
6996448	–1.07	–0.09	1.29	0.37	tropomyosin 1, alpha	*Tpm1*
6921068	–1.08	–0.11	1.32	0.40	Dynactin 3 | AT rich interactive domain 3C (BRIGHT-like)	*Dctn3|Arid3c*
6785483	–1.17	–0.22	1.32	0.40	RAS-related C3 botulinum substrate 3	*Rac3*
6818956	–1.15	–0.21	1.33	0.42	Ribonuclease, RNase A family 4 | angiogenin, ribonuclease, RNase A family, 5	*Rnase4|Ang*
6784526	1.06	0.08	1.35	0.43	Myosin, light polypeptide 4 | lin-52 homolog (C. elegans) | predicted gene 7020	*Myl4|Lin52|Gm7020*
6934584	–1.08	–0.11	1.40	0.48	RAN, member RAS oncogene family	*Ran*
6817978	–1.05	–0.07	1.42	0.51	Troponin C, cardiac/slow skeletal	*Tnnc1*

FC: Fold Change; Red: Upregulated genes; Blue: downregulated genes.

## Discussion

Although, it is now well-known that GLA is structurally related to glutamate and clearly neurotoxic, its putative aversive effects on neuroblast homeostasis (for which glutamate is of crucial value) remained unexplored. Our recent study suggested that behavioral alterations induced by perinatal exposure to GLA could be related to neurogenesis disturbances due to altered brain expression of relevant genes, like *Pten*, well-known to be importantly involved in this process (Laugeray et al., 2014). Herein, we tested this hypothesis thanks to both *in vivo* and *ex vivo* analyses.

### SVZ as a potential open window to GLA neurotoxicity

It is now well-established that the neurotransmitters GABA and glutamate are of crucial importance during neuronal migration from the SVZ to the OB and, as such, are likely to contribute to the pathogenesis of neuronal migration disorders (Platel et al., 2010). Here, we assumed that GLA, as an analog of glutamate, can affect cell proliferation and neuroblast migration by interfering with glutamate signaling, and thus with SVZ functions. In agreement with this hypothesis glutamate homeostasis is known to be importantly involved in RMS migration processes (Platel et al., 2008). However further experiments are needed to support this statement. We were able to show that exposure to low dose GLA (GLA0.2) led to neuroblast accumulation in the SVZ *in vivo* at PDN15. Such an alteration has already been reported in some ASD patients (Wegiel et al., 2010). Together with our data, these findings strengthen our hypothesis of a link between GLA exposure, abnormal neurogenesis in the SVZ and ASD. Interestingly, at the highest dose, GLA did not affect SVZ thickness but, rather, led to ectopic migration of neuroblasts outside the SVZ. These data suggested that the SVZ, lining the walls of the lateral ventricles, is sensitive to exogenous toxicants, and subsequently may constitute the real “window of the brain” as proposed by many authors (Gross and Weindl, 1987; Moyse et al., 2006; Joly et al., 2007; Lin et al., 2015). Such an assumption is reinforced by a recent study showing that perinatal exposure to another environmental toxicant, methotrexate, also led to SVZ alterations (Hirako et al., 2016). These aversive effects of exogenous compounds on the SVZ could be inherent to its intrinsic structure characterized by permeable fenestrated capillaries and thus a lack of endothelial blood–brain-barrier (BBB) (Johnson and Gross, 1993; Tavazoie et al., 2008). Consequently, the SVZ stem cell niche is in the front line to respond to systemic xenobiotics and is thus likely to be extremely sensitive to environmental toxicants.

### Impaired proliferation, migration, and apoptotic processes after perinatal exposure to GLA

Activation of glutamate receptors causes transient increases in intracellular Ca^2+^ promoting neuronal migration by acting on the cytoskeleton protein regulators (Luhmann et al., 2015). Even if our data showed that perinatal exposure to two doses of GLA negatively impacts neuroblast homeostasis within the SVZ-OB system, the present results also show that GLA is able to induce two different patterns of SVZ alterations suggesting that several targets might be impacted depending on the dose. Indeed, cell morphology, considered to be pivotal in the process of migration (Luskin, 1993; Alvarez-Buylla and Garcia-Verdugo, 2002), is differentially affected by GLA depending on the dose as illustrated by *ex vivo* SVZ explants where GLA0.2-exposed neuroblasts display highly compacted chains whereas neuroblasts from GLA1-exposed explants seem less altered in their morphology (although still different from CTL ones). In support of this, our *in vivo* BrdU labeling experiments also show differential effects of GLA depending on the dose. In agreement with the literature, we observe in CTL offspring that, BrdU+ cells go out from the SVZ and migrate through the RMS to finally reach the OB 2 days later. In GLA0.2-exposed animals, BrdU+ neuroblasts are able to migrate from the SVZ to the OB but our results indicate that changes in SVZ cell proliferation and defects in migration processes significantly delay the temporal dynamics of neuroblast migration On the contrary, no alteration of proliferation is observed in GLA1-exposed offspring *in vivo* while many cells, despite being able to leave the SVZ as well as CTL ones, are found in an abnormal ectopic location, the *caudate putamen* (Cpu), a situation never seen in CTL neuroblasts. These results are consistent with the SVZ enlargement observed *in vivo* and the compacted aspect of chains in SVZ explants, only seen at the lowest dose. Therefore, it is of importance to notice that, for some parameters, the doses have the same effect while for others the effect is qualitatively different. Moreover, we find the OB in GLA0.2 and GLA1-exposed mice to have an apparently normal size and structure. This point is relevant because the number of cells reaching the OB is thought to regulate the size of the bulbs (Gheusi, 2000). At first glance, the decreased number of TUNEL^+^ cells in GLA0.2 and GLA1-exposed mice seem at odds with the lower increase in BrdU^+^ cells and the normal bulb morphology. These data are however in line with previous study reporting neuronal migration disorders following *in utero* exposure to several environmental factors in humans and in animal models (Wisniewski et al., 1983; Miller, 1986; Gressens et al., 1992; Shinmura et al., 1997).

### Reelin and cytoskeleton alterations as the cause of GLA-induced neurogenesis defects

Reelin expression is essential for neuronal migration in the developing brain, acting as a “detachment signal” of postnatal neuroblast from the RMS (Simo et al., 2007). In GLA-exposed mice, the OB expression pattern of reelin seem to be altered irrespective of the dose. Such findings are in accordance with our *in vivo* and *ex vivo* experiments showing disturbances of neuroblast migration. The reelin pathway corrects the migration of early generated interneurons within the olfactory bulb. This function is a prerequisite for correct OB lamination (Hack et al., 2002; Hellwig et al., 2012). GLA-induced alterations of the laminar organization within the OB are clearly consistent with this. In support of this, reeler mice have been shown to exhibit ectopic accumulation of neuroblasts in the RMS, failing to transit from tangential migration in the RMS to radial migration in the OB (Ayala et al., 2011; Sun et al., 2010). Interestingly, complex interactions between reeler genotype and early exposure to environmental toxicants have already been demonstrated, (Keller and Persico, 2003; Laviola et al., 2006; Persico and Bourgeron, 2006; Mullen et al., 2013).

GLA-induced neurogenesis defects was also reinforced by observing our transcriptomic analyses on PND15 brains perinatally exposed to GLA. Indeed, expression pattern of a series of genes regulating the cytoskeleton, cell proliferation and cell migration were affected by GLA exposure. Interestingly, GLA0.2 and GLA1 groups present a different deregulated pattern, and no dose effect was observed. This result was in accordance with our *ex vivo* data showing that a different cellular morphology observed in explants experiments treated with GLA0.2 or GLA1. Therefore, GLA0.2 and GLA1 specifically induced gene deregulation explaining the compacted neuroblasts chains morphology and abnormal morphology with extension and branching of the growth cone and abnormally long dendrites, respectively. However, 11 genes were deregulated in both GLA0.2 and GLA1 treated groups. Interestingly, these commonly deregulated genes included pivotal representatives for the major processes controlling cell migration such as cytoskeleton rearrangements (*Rac, Rho, Rock*), cell adhesion (*Arp2/3, Pfn, Actn, Erm*), and chemotactic signalization (*Rtk, Itg, Ras*) (Khodosevich et al., 2009). ARP complex *Arpc3 and Arpc4* which nucleates actin filament growth from the minus end and allows rapid elongation at the plus end (Alberts et al., 2002), PFN (*Pfn1 and Fscn1*) promoting actin migration/proliferation of non-muscle cells (Wang et al., 2014), MLC (*My19)* regulating cytoskeleton contraction (Saban et al., 2006), GF (*FgF1*) regulating cytoskeletal organization collagen contraction (Ding et al., 2000), GPCR/Chemokine (*Chrm4* and *CCl3*) regulating chemotaxis cell migration and actin cytoskeleton moving machinery (Yang et al., 2012), and *Ras/Rac* system (*Arhgdib*) which controls the assembly and disassembly of the actin cytoskeleton in response to extracellular signals (Ory et al., 2000). RhoGTPase family is one of the major regulators of cytoskeletal properties and plays essential functions in cerebral cortex development. These functions are known to be highly associated with glutamate homeostasis to regulate neuroblast proliferation and migration (Di Giorgi-Gerevini et al., 2005; Platel et al., 2008). Here we show, that perinatal organophosphate GLA exposure affect cell migration modulating not only Reelin activity, but also a series of other genes involved in cytoskeleton regulation, thus potentially contributing to the neurodevelopmental basis of autism-like behavior.

### On the unusual “dose-dependent” effects of perinatal exposure to GLA

Most of the results presented here indicate that perinatal exposure to GLA may have different effects depending on the dose to which individuals are exposed. In support of this, it is of interest to see that the lowest dose seems to be more harmful in regards of some parameters we studied here. Such results clearly challenge the prevailing dogma that “the dose makes the poison” as the reported effects show a qualitatively different dose-dependent response. One explanation for this could be related to the structural analogy of GLA to the excitatory neurotransmitter glutamate. Indeed, GLA may have a glutamate-like effect at low dosage whereas, at higher dosage, it may induce detrimental effects on neurons irrespective of its signaling to glutamate receptors. For instance, one can assume that, at high dosage, GLA may be added to α/β tubulin heterodimers instead of glutamate and consequently have disturbing effects on polyglutamylation processes. Some experiments are currently in progress in order to test such a hypothesis.

For a number of years now, there have been reports showing that environmental toxicants can have effects at low dosage and that these cannot be predicted and extrapolated from effects at higher doses (Vandenberg et al., 2012). The present data strongly suggest that GLA is likely to be one of these toxicant. Our previous study (Laugeray et al., 2014) considerably strengthens this assumption as some neurodevelopmental outputs were commonly affected by the two doses of GLA while for others, it was not the case. This was especially true for anatomical abnormalities indicating macrocephaly at 1 mg/kg and microcephaly at 0.2 mg/kg. Interestingly, such changes were concomitant with reduced *Pten* mRNA levels in the brain of GLA1-exposed pups matching the macrocephaly in the same mice, but perinatal exposure to the lower dose had the opposite effect on both *Pten* expression and brain size (microcephaly). Such a non-classical “dose dependent” effects were also observed on bio-behavioral parameters (Laugeray et al., 2014). It is therefore essential to build up our knowledge of possible harmful effects of low-level perinatal exposure to pesticides. This is a relevant and topical issue as many governmental reports have noted that early exposure (pre and postnatal) to low or very low doses of pesticides is not usually covered by the tests required for regulatory approval, and therefore that it is impossible to estimate such adverse effects (Bonnefoy, 2012; Watts, 2012).

In summary, our work demonstrates for the first time the deleterious effect of perinatal exposure to GLA results in abnormal brain development, both at the cellular and molecular levels, providing a putative structural explanation for GLA-induced ASD-like phenotypes in mice. Our data not only identify the SVZ as a novel target for environmental toxicants, in particular in case of early exposure to low doses but also pave the way for unraveling the molecular events that orchestrate the effect of GLA on cell cytoskeleton.

## Author contributions

Research was designed by AH and SM. Research was realized by AH, AL, JF, OR, and OP. Research was analyzed and discussed by AH, AM, JP, OP, and SM. Paper was written by AH, CM, VQ, OP, and SM.

## Funding

This work was supported by the French National Research Agency – ANR (CESA-10-007 – NEUROPEST), and Region Centre (Doctoral fellowship to Ameziane Herzine).

### Conflict of interest statement

The authors declare that the research was conducted in the absence of any commercial or financial relationships that could be construed as a potential conflict of interest.

## Supplementary material

Supplementary Figure 1 **Effect of prenatal exposure to GLA on cyto-architecture of the olfactory bulbs. (A)** Schematic of a coronal section of the olfactory bulb of a mouse brain. **(B)** Coronal sections in mouse olfactory bulbs of CTL, GLA0.2, or GLA1 stained with DCX antibody (green) and DAPI (blue), mice bulbs CTL has a characteristic architecture and many parallel neuroblasts which migrate to the external layers of the olfactory bulbs; Bulbs from exposed mice have a different architecture from that of CTL mice, neuroblasts migration no longer form a parallel architecture. Scale bar 50 μm. NE, neuroepithelium; Mi, mitral layer; GrL, Granular Layer.

Supplementary Figure 2 **Example of analyzing of arrival of neuroblasts to the olfactory bulbs by measuring the integrated density**. PND5 pups were injected with BrdU. Coronal sections of the CTL OB were stained with BrdU (green) and counterstained with DAPI (blue) 2 h (T0+2 h) or 2 days (T0+2 D) after BrdU injection **(top panel)**. Measurement of the Integrated intensity (RawIntDen) was performed with imageJ software in three OB sub-structural region (neuroepithelium:NE; granular layer: GrL; and glomerular layer: Glo). At T0+2 h all proliferating cells are BrdU+, the labeling is homogeneous in the differents layers of the olfactory bulbs. At T0+2 D, a large number of BrdU+ cells arrive in the OB. The measurements of the integrated density showed an increase of signal relative to the number of BrdU+ cells **(bottom panel)** showing neuroepithelia (ne) neuroblasts arrival from rostral stream at T0+2 D. Scale bar: 100 μm. NE, neuroepithelium; GrL, Granular Layer; Glo, Glomerular layer.

Supplementary Figure 3 **Morphology changing of neuroblast *ex vivo* explant culture**. Some neuroblasts migrate out of chain. **(A)** In control, these neuroblasts have a classical morphology with short cell body, a clear bipolar morphology and a short and condensed growth cone. **(B)** In GLA1 exposed explants, free neuroblasts display abnormal morphology with long dendrite processes and unstructured growth cone (arrow) displaying extensive branching. Scale bar: 20 μm.
