# Supplementary figures and images for: Perinatal Exposure to Glufosinate Ammonium Herbicide Impairs Neurogenesis and Neuroblast Migration through Cytoskeleton Destabilization
Source: Front Cell Neurosci. 2016 Aug 9;10:191. doi: 10.3389/fncel.2016.00191 (PMC4977287; doi:10.3389/fncel.2016.00191)

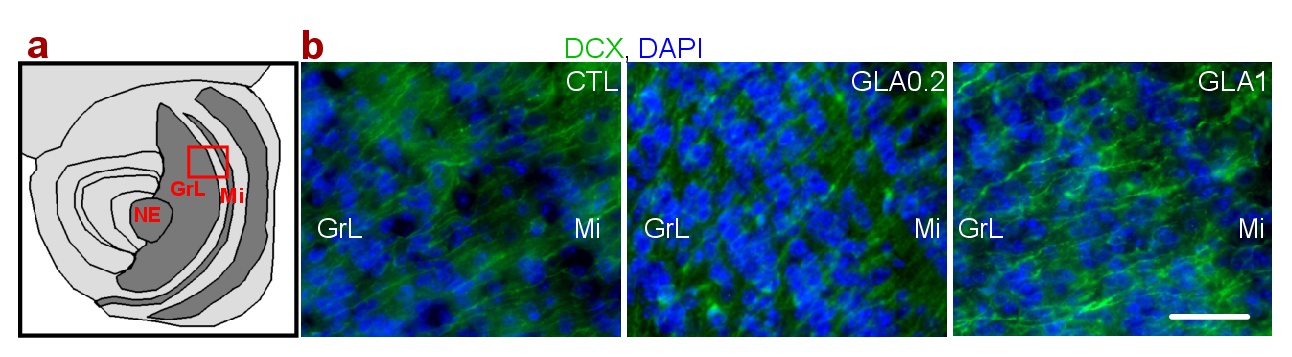

Supplement: Supplementary Figure 1 — Effect of prenatal exposure to GLA on cyto-architecture of the olfactory bulbs. (A) Schematic of a coronal section of the olfactory bulb of a mouse brain. (B) Coronal sections in mouse olfactory bulbs of CTL, GLA0.2, or GLA1 stained with DCX antibody (green) and DAPI (blue), mice bulbs CTL has a characteristic architecture and many parallel neuroblasts which migrate to the external layers of the olfactory bulbs; Bulbs from exposed mice have a different architecture from that of CTL mice, neuroblasts migration no longer form a parallel architecture. Scale bar 50 μm. NE, neuroepithelium; Mi, mitral layer; GrL, Granular Layer. [file Image1.JPEG]

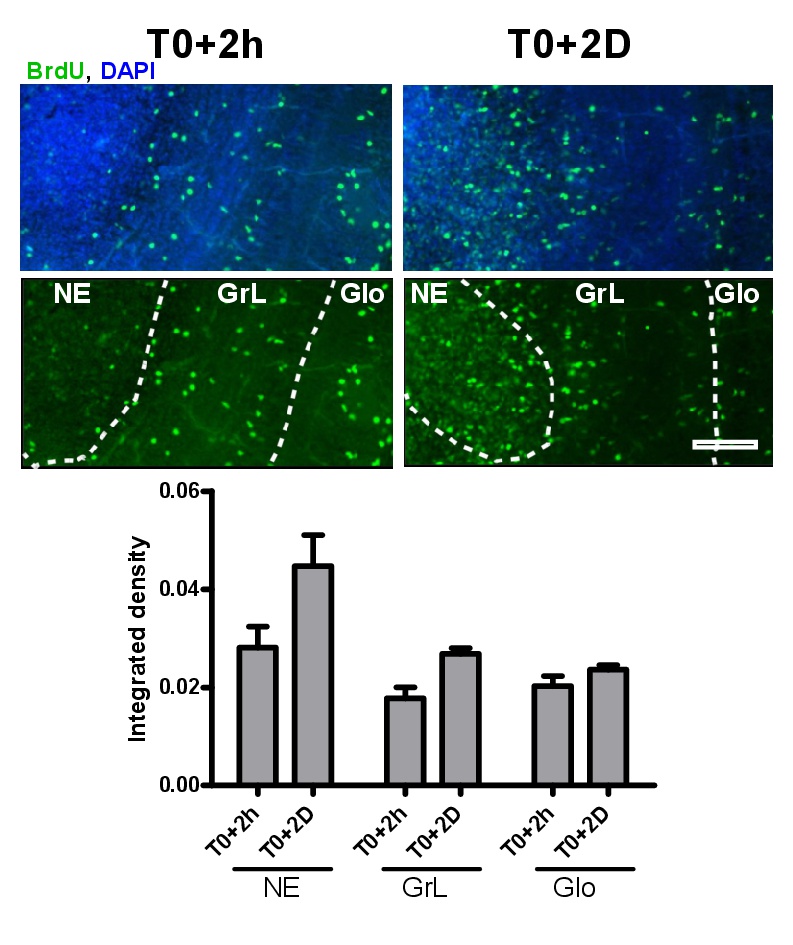

Supplement: Supplementary Figure 2 — Example of analyzing of arrival of neuroblasts to the olfactory bulbs by measuring the integrated density. PND5 pups were injected with BrdU. Coronal sections of the CTL OB were stained with BrdU (green) and counterstained with DAPI (blue) 2 h (T0+2 h) or 2 days (T0+2 D) after BrdU injection (top panel). Measurement of the Integrated intensity (RawIntDen) was performed with imageJ software in three OB sub-structural region (neuroepithelium:NE; granular layer: GrL; and glomerular layer: Glo). At T0+2 h all proliferating cells are BrdU+, the labeling is homogeneous in the differents layers of the olfactory bulbs. At T0+2 D, a large number of BrdU+ cells arrive in the OB. The measurements of the integrated density showed an increase of signal relative to the number of BrdU+ cells (bottom panel) showing neuroepithelia (ne) neuroblasts arrival from rostral stream at T0+2 D. Scale bar: 100 μm. NE, neuroepithelium; GrL, Granular Layer; Glo, Glomerular layer. [file Image2.JPEG]

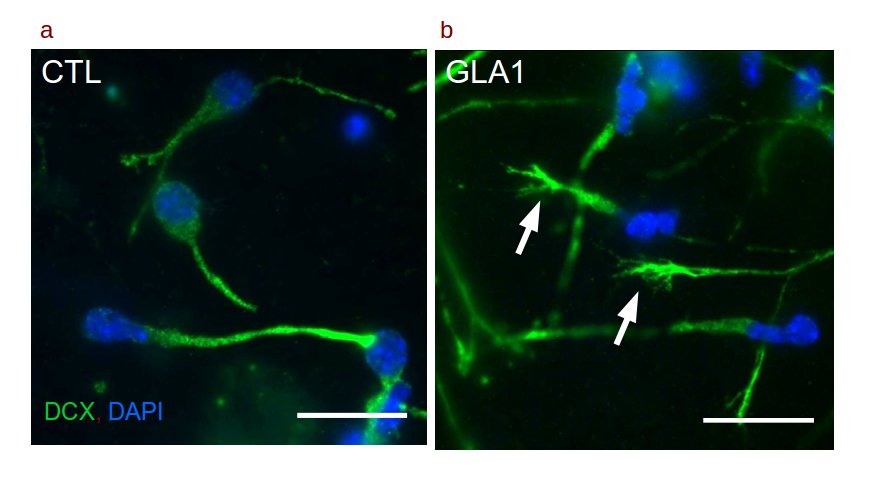

Supplement: Supplementary Figure 3 — Morphology changing of neuroblast ex vivo explant culture. Some neuroblasts migrate out of chain. (A) In control, these neuroblasts have a classical morphology with short cell body, a clear bipolar morphology and a short and condensed growth cone. (B) In GLA1 exposed explants, free neuroblasts display abnormal morphology with long dendrite processes and unstructured growth cone (arrow) displaying extensive branching. Scale bar: 20 μm. [file Image3.JPEG]
